# Clean the Ni-Rich Cathode Material Surface With Boric Acid to Improve Its Storage Performance

**DOI:** 10.3389/fchem.2020.00573

**Published:** 2020-07-24

**Authors:** Yuefeng Su, Gang Chen, Lai Chen, Linwei Li, Cong Li, Rui Ding, Jiahui Liu, Zhao Lv, Yun Lu, Liying Bao, Guoqiang Tan, Shi Chen, Feng Wu

**Affiliations:** ^1^Beijing Key Laboratory of Environmental Science and Engineering, School of Materials Science and Engineering, Beijing Institute of Technology, Beijing, China; ^2^Beijing Institute of Technology Chongqing Innovation Center, Chongqing, China; ^3^School of Materials Science and Engineering, Beijing Institute of Technology, Beijing, China

**Keywords:** Ni-rich materials, surface washing, boric acid, residual lithium compounds, storage performance

## Abstract

The existence of residual lithium compounds (RLCs) on the surface of layered Ni-rich materials will deteriorate the electrochemical properties and cause safety problem. This work presents an effective surface washing method to remove the RLCs from LiNi_0.90_Co_0.06_Mn_0.04_O_2_ material surface, *via* ethyl alcohol solution that contains low concentration of boric acid. It is a low-cost process because the filter liquor can be recycled. The optimal parameters including washing time, boric acid concentration, and solid–liquid ratio were systematically studied. It has been determined by powder pH and Fourier transform infrared spectra results that the amount of RLCs was reduced effectively, and the storage performance was significantly enhanced for the washed samples. The 150th capacity retentions after storing had increased from 68.39% of pristine material to 85.46–94.84% of the washed materials. The performance enhancements should be ascribed to the surface washing process, which removed not only the RLCs, but also the loose primary particles effectively.

## Introduction

Nowadays, lithium-ion batteries (LIBs) have been considered as the most feasible equipment to utilize the electrical energy. However, it is still urgent to develop high-energy density LIBs to satisfy the ever-growing demands of electric vehicles (He et al., 2018; Su et al., 2018a; Kong et al., 2019; Liang et al., 2019). The cathode materials have been considered as the focus and weak link of high-energy LIBs; thus, the high-capacity layered Ni-rich LiNi_*x*_Co_*y*_Mn_1-x-y_O_2_ (0.5 < *x* < 1, 0 < *y* < 0.5) materials emerged and had been studied widely as one of the most promising cathode materials in recent years (Lin et al., 2014; Wu et al., 2018, 2019; Qiu et al., 2019).

However, although increased nickel content in Ni-rich cathode materials enables them to deliver higher capacity, more residual lithium compounds (RLCs) will be formed on their surface. The RLC mainly contains Li_2_O, Li_2_O_2_, LiOH, LiHCO_3_, and Li_2_CO_3_ (Liu et al., 2004; Kim et al., 2006; Bichon et al., 2019; Xu S. et al., 2019; Zhang et al., 2019), which origins from the vestigial of lithium salts and spontaneous reduction reaction of Ni^3+^ during the synthesis and storage process (Junhyeok et al., 2018). In detail, excess 5 mol% lithium salts are usually applied to compensate the evaporation loss during high-temperature calcination process (Liang et al., 2017). The obvious side effect, however, is that in the event of an inadequate calcination process, it will bring excessive lithium salts (e.g., LiOH and Li_2_CO_3_), and the undecomposed lithium compound intermediates (like Li_2_O and Li_2_O_2_) that linger on the surface of Ni-rich cathode materials. Especially, with higher nickel content (especially >60%), the calcination temperature needs to be lower and thus more likely leading to the formation of RLC (Junhyeok et al., 2018; Park J. -H. et al., 2018). On the other side, the surface active lattice lithium and oxygen species of Ni-rich cathode materials would react with the H_2_O and CO_2_ in the air during storage under the influence of reduction of sensitive Ni^3+^, forming the undesirable RLC and surface NiO rock-salt layer (Huang et al., 2019).

This undesired RLC has many adverse effects on the Ni-rich materials. First, RLC would absorb the trace water in the air and increase the powder alkalinity, which will further gel the slurry during electrode fabrication process, corrode the current collector, and worsen the consistency of electrode and the corresponding batteries (He et al., 2018; Junhyeok et al., 2018; Bichon et al., 2019). Second, this insulating RLC would also react with the electrolyte and produce gas such as CO_2_, thus deteriorating the electrochemical performances and causing safety problem (Hatsukade et al., 2018). Third, Grenier et al. (2017) had proved that the existence of Li_2_CO_3_ layer on the surface of Ni-rich materials will induce a “two-phase” behavior during the initial charge process, which severely hinders the exploration of the intrinsic reaction mechanism of Ni-rich materials. Last but not least, the formation of RLC during the storage consumes the lithium inventory, which will cause deteriorative electrochemical performance.

The most common way to reduce the RLC is water washing (Kimijima et al., 2016; Li et al., 2018). Unfortunately, although this treatment can remove the RLC on the surface of materials, it cannot prevent the treated materials from keeping reacting with water and carbon dioxide in the air. More seriously, the water washing process will worsen the thermal stability of the Ni-rich materials at some extent and make the washed materials be more sensitive to the air (Xiong et al., 2013). As a result, some improved strategies have emerged; for instance, Xu et al. (2017) used protonated polyaniline (PANI) in *N*-methyl pyrrolidone solvent to wash the LiNi_0.8_Co_0.1_Mn_0.1_O_2_ material. The H^+^ in the solvent reacted with the RLC, whereas the remained conductive PANI formed a protective layer on LiNi_0.8_Co_0.1_Mn_0.1_O_2_ surface after washing. Liu et al. (2018) adopted the pure ethanol solvent instead of water to wash the LiNi_0.8_Co_0.15_Al_0.05_O_2_ materials. These results show that washing method is indeed an effective way to reduce the amount of RLC, whereas the ethanol solvent can further enhance the material resistance to the humidity in the air.

**Figure 1:**
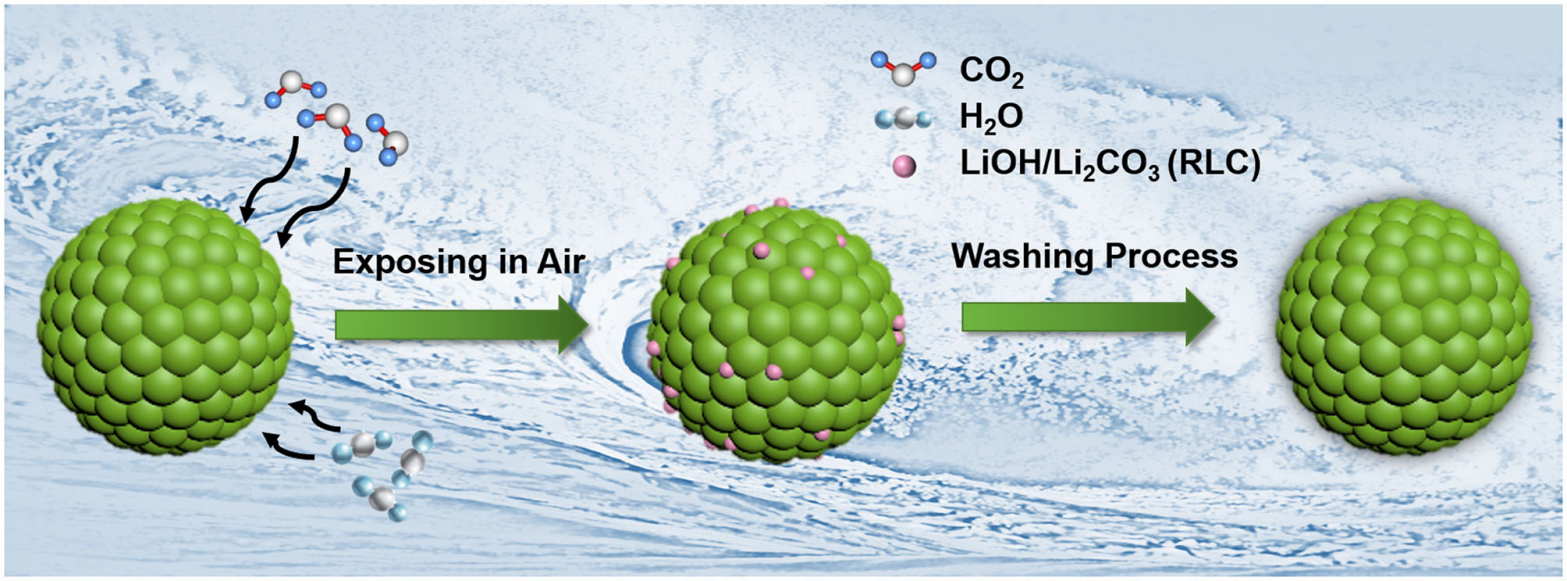
The schematic diagrams for the washing process of LiNi_0.90_Co_0.06_Mn_0.04_O_2_ materials.

Here we propose a universal surface washing method *via* ethanol solvent with appropriate amount of boric acid (BA) for Ni-rich LiNi_0.90_Co_0.06_Mn_0.04_O_2_ cathode material. Boron element has been widely used to modify the Ni-rich cathode materials (Hu et al., 2017; Lv et al., 2017; Chen et al., 2018; Park K. -J. et al., 2018), whereas BA can react with RLC easily without bringing harmful effects because it is a weak acid. Besides, the ethanol solvent also could remove the RLC. The schematic diagram for the washing process is shown in Figure 1. During this process, the filtrate can be recycled and used to wash the cathode materials again, which lowers the cost. The key parameters of this washing method, including washing time, concentration of BA, and solid–liquid ratio of LiNi_0.90_Co_0.06_Mn_0.04_O_2_ material to ethyl alcohol solution, had been investigated systematically in this work. The testing results show that benefiting from the washing process, the pH values of treated materials were reduced effectively. Accordingly, their electrochemical performances, especially the short-time storage performances, had been significantly enhanced. Overall, our work provides a promising strategy for treating the RLC on Ni-rich material surface with lower cost and simple steps.

## Experimental Section

### The Preparations of Pristine and Washed Materials

The LiNi_0.90_Co_0.06_Mn_0.04_O_2_ was prepared *via* a coprecipitation method with solid-state reaction method according to our previous report (Su et al., 2018a), and the details are exhibited in the Supporting Information. The pristine sample was labeled as “BA-0” and used for further modifying experiments. To prepare the washed materials, some amount of BA was dissolved in pure ethyl alcohol to form a clear washing liquid first. Then 1 g of the freshly prepared powders was dispersed in the ethanol solvent and stirred for several minutes. After the washing stage, the powders were filtered, and the filtrate was collected for reusing. The parameters of washing process, labeled as washing time, concentration of BA, and solid–liquid ratio of LiNi_0.90_Co_0.06_Mn_0.04_O_2_ material to ethyl alcohol solution, are summarized in Table 1, and their corresponding samples were labeled as BA-1 to BA-9. After the filtration, the powders were stored in the air randomly. Before fabricating the cathode electrodes, all the powders, including BA-0, were dried up at 80°C for 1 h and then sintered at 400°C for 2 h under oxygen atmosphere to remove all possible ethyl alcohol remained on the NCM particle surface and rule out the influences of reheat process.

**Table 1:** Summary of the treat parameters and Li/Ni/Co/Mn molar ratios calculated from ICP-OES of all the samples.

**Sample no**.	**Solid–liquid ratio (g:mL)**	**Washing time (min)**	**Concentration of BA (g/L)**	**Li/Ni/Co/Mn calculated from ICP**
BA-0	0	0	0	100.5:90.1:5.9:4.0
BA-1	1:1	5	0.25	100.3:89.9:6.0:4.1
BA-2	1:1	10	0.5	100.1:90.0:6.1:4.0
BA-3	1:1	20	1	99.9:89.8:6.1:4.1
BA-4	1:5	5	1	100.0:90.1:6.0:3.9
BA-5	1:5	10	0.5	100.0:90.0:6.0:4.0
BA-6	1:5	20	0.25	100.0:90.0:5.9:4.1
BA-7	1:10	5	0.25	100.3:89.9:6.0:4.1
BA-8	1:10	10	1	99.8:90.2:5.8:4.0
BA-9	1:10	20	0.5	100.1:90.1:6.0:3.9

### Characterizations of the Materials

The compositions, morphologies, and crystalline structures of all samples were detected by inductively coupled plasma optical emission spectrometry (ICP-OES; Agilent ICPOES730, USA), powder X-ray diffraction (XRD; Rigaku UltimaIV-185 instrument, Japan), X-ray photoelectron spectroscopy (XPS; ULVAC-PHI, Inc., Japan), field-emission scanning electron microscopy (SEM; FEI Quanta 250 instrument, USA), and high-resolution transmission electron microscopy (HR-TEM; JEOL JEM-2100 instrument, Japan). The pH values were confirmed by a Mettler Toledo FE20 pH meter (Mettler Toledo, Switzerland), while the existence of RLC was proven by Fourier transform infrared spectra (FT-IR; Nicolet IS10, USA).

### Electrochemical Measurements

All the samples were assembled into CR2025 coin-type half cells, which consist of lithium metal anode and a commercial Celgard 2500 separator. The manufacturing details of cathode electrode and cells were kept the same with our previous work (Su et al., 2019). The electrochemical cycling tests (2.75–4.3 V, vs. Li^+^/Li) and the potentiostatic intermittent titration technique (PITT) experiments of cells were operated on the CT2001A Land Instruments (Wuhan, China) at room temperature. Electrochemical impedance spectroscopy (EIS) and measurements were operated at CHI660E electrochemical workstation (Shanghai, China).

## Results and Discussion

The compositions of all the samples were tested by ICP-OES, and the results are listed in Table 1. The similar molar ratio values, as we expected, reveal that the faintly acid BA had not damaged the samples as the transition metal elements were not leached out after washing. Note that the lithium contents of the washed materials are decreased with the increasing washing time and BA concentration. The cause of reduced amount of lithium needs further confirmation, which may originate from the reduction of RLC or the dissolution of lattice lithium from H^+^/Li^+^ ion exchange.

**Figure 2:**
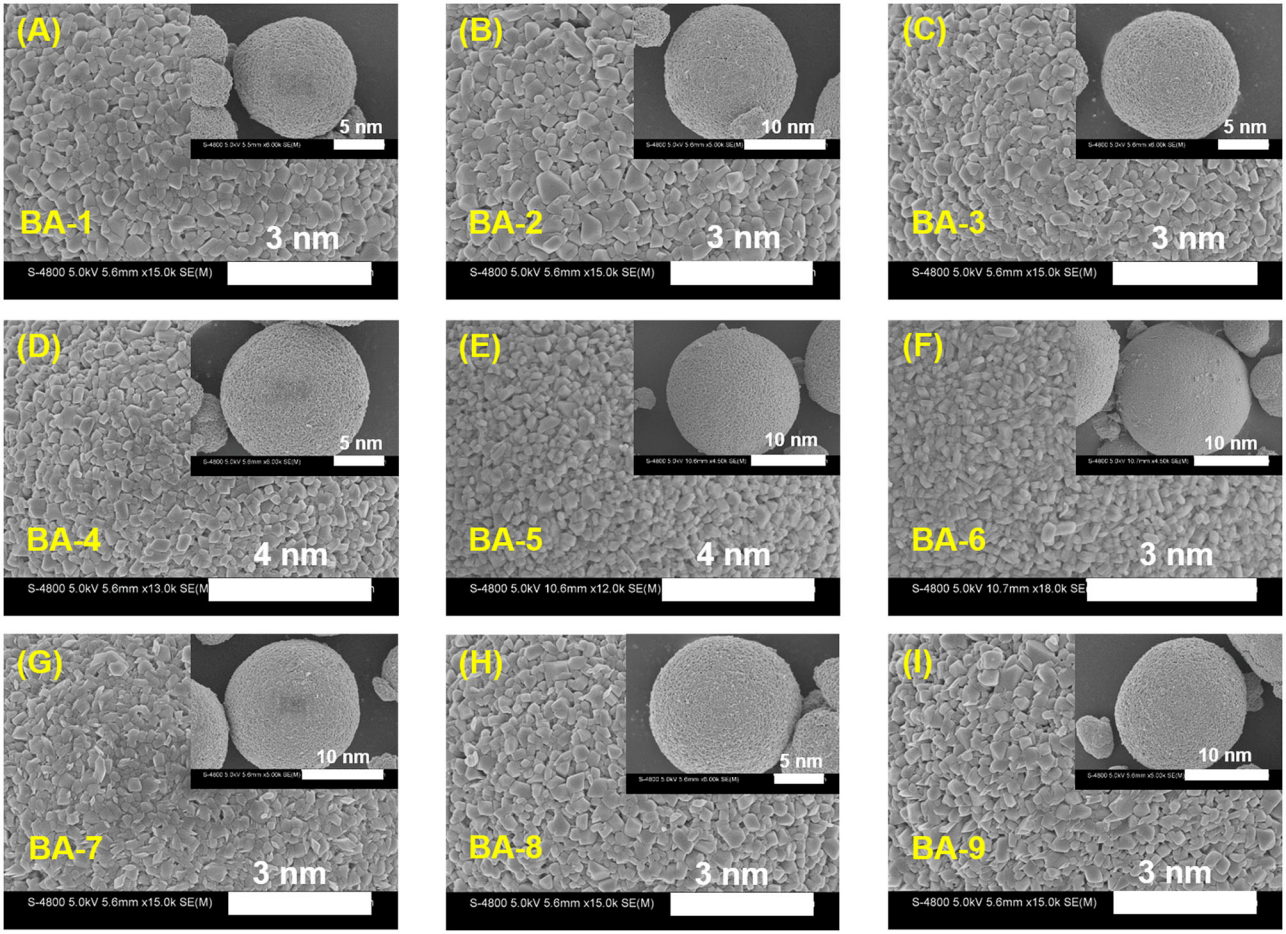
The magnifying SEM images of **(A–I)** BA-1 to BA-9. The inserts are SEM images of their corresponding particles.

To further ascertain the influences of washing process to Ni-rich materials, the morphologies of all the washed samples were observed by SEM images as exhibited in Figure 2, whereas that of BA-0 is shown in Figure S1. The secondary particles exhibit similar morphology, but the magnified inserts indicate that there are slightly differences in primary particles. Not only that the primary particles size of the washed materials seems larger, but also their surface becomes clearer than those of pristine material. The larger particle size may result from the introduction of boron element (Chen et al., 2018), whereas the cleaner surface may ascribe to falling off of RLC (Xu S. et al., 2019).

**Figure 3:**
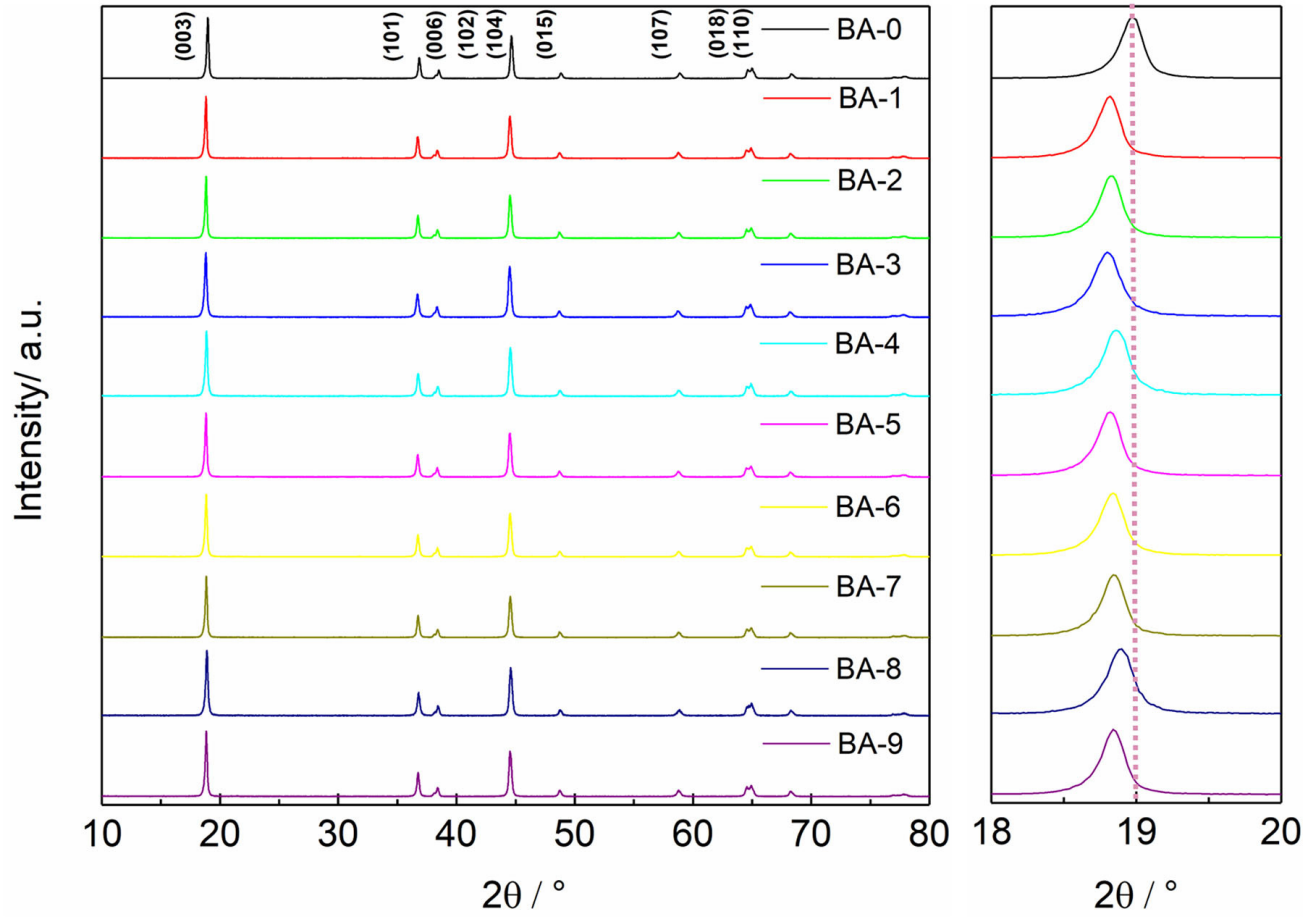
The whole and detailed XRD patterns of all samples.

The surface changes in morphology are generally accompanied by the changes in lattice structure. The XRD patterns exhibited in Figure 3 reveal that all samples belong to a hexagonal α-NaFeO_2_ structure with an R$\bar{3}$m space group (Xie et al., 2019; Su et al., 2020). The clear splitting between (006)/(102) peaks and (018)/(110) peaks of all samples proves that the materials have a well-ordered layered structure, whereas the BA or ethyl alcohol has not deteriorated the bulk structure of Ni-rich materials. Another comforting phenomenon is that all XRD patterns show no extra impurity peaks, indicating that the contents of impurity phases such as RLC or the possible H_3_BO_3_-based materials are absent or very low (Xu G. -L. et al., 2019). The ratios of *I*_(003)_/*I*_(104)_ of all samples were also calculated, and the results are listed in Table S1. Although the ratio values of some samples are reduced, all the calculated values are still higher than 1.2, meaning the low cation mixing degree (Zhang et al., 2018; Weigel et al., 2019). The changed *I*_(003)_/*I*_(104)_ values of the washed samples indicate the washing process may affect the lattice structure. To further investigate the detailed structure changes after washing, the (003) peaks of all samples are magnified as shown in Figure 3 and Table S1. Comparing with the peak position of the BA-0 sample, the (003) peaks of all washed materials shift to a lower angle in varying degrees, which manifests larger interplanar spacings (Wang et al., 2016). The larger interplanar spacing may result from the dissolution of lattice lithium during the washing process, which consists with the ICP-OES results.

**Figure 4:**
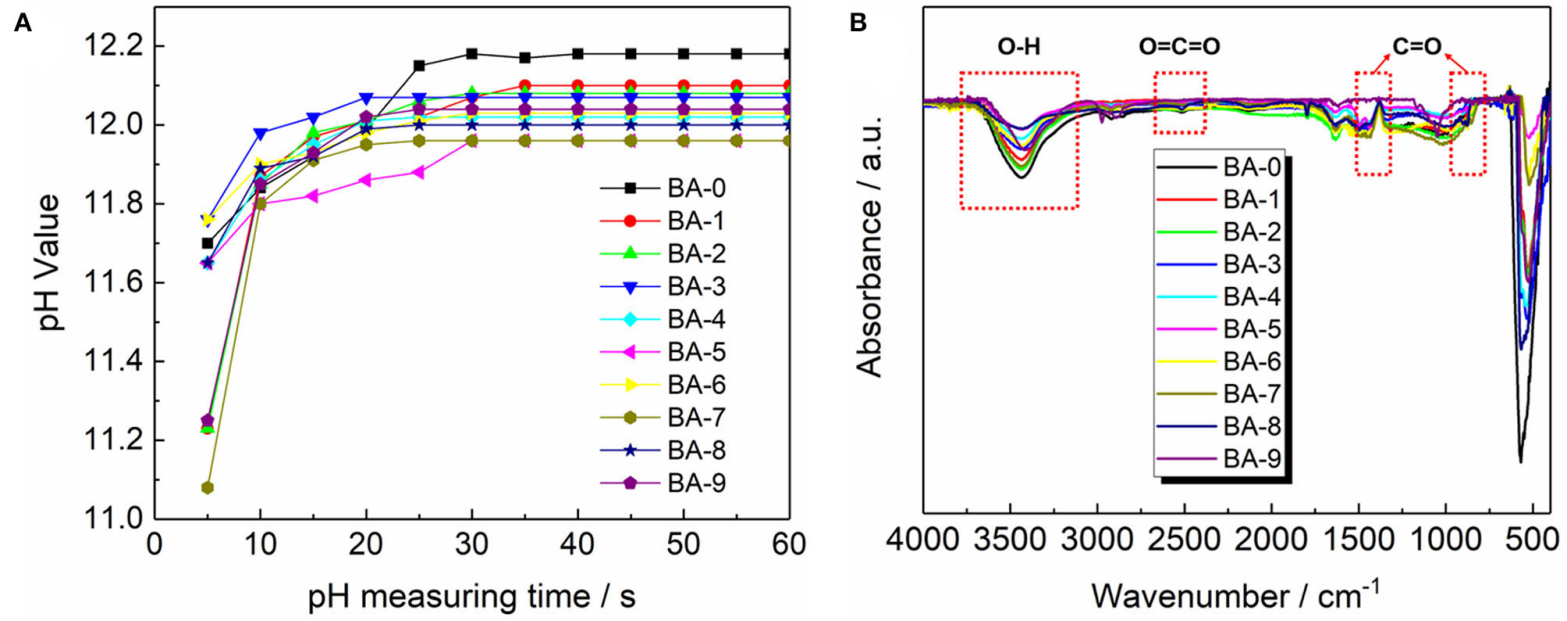
**(A)** Plot of pH vs. the pH measuring time and **(B)** the FT-IR spectra of all the samples.

The pH measurements were adopted to check if the RLC had been removed from the samples surface. The pH value curves are presented in Figure 4A. All the pH curves in Figure 4A exhibit an increasing tendency followed by stable values. The increase in pH value at the first 30 s may ascribe to the dissolution of alkaline LiOH (Xiong et al., 2013), and the final pH value of BA-0 is much higher than all those of the treated materials. Because the pH value could reflect the total amount of RLC on the surface of materials as mentioned previously, therefore it is indubitable that the washing process we proposed could reduce the amount of RLC effectively.

To further confirm the content of the RLC on the samples surface, the FT-IR test was applied, and the results in Figure 4B give more evidence of the surface changes. The peak at 3,200–3,650 cm^−1^ belongs to the O-H stretching vibration from LiOH, whereas the other two bands at 870 and 1,450 cm^−1^, respectively, originated from the C=O and O=C=O vibration in Li_2_CO_3_ (Xiong et al., 2013). All the FT-IR patterns demonstrate stronger signal of LiOH than that of Li_2_CO_3_, which means there are more LiOH on the surface of samples before storage. Meanwhile, compared with the BA-0, the washed materials show significantly decreased peak intensities of LiOH, indicating an effective removal of RLC *via* our washing process.

The washing process may influence the valence states of elements and microstructures of Ni-rich materials. Therefore, the XPS measurements and HR-TEM analysis were applied to the BA-0 and BA-7 samples to verify the washing effects and structural changes, and the results are shown in Figure S2. Figures S2A–D exhibits the XPS spectra of BA-0 and BA-7, proving the similar binding energies of two samples and the same valence states of Co and Mn. It should be noted that the binding energies of Ni 2p_3/2_ in Figure S2B are different, which originated from the different content ratios of Ni^2+^/Ni^3+^ in these samples. The Ni^2+^/Ni^3+^ ratio value decreases from 0.52 to 0.33 after washing process, and the reduction of Ni^2+^ implies that there might be structural variations in Ni-rich materials, which could be confirmed by the HR-TEM tests. As can be seen from Figures S2E,F, the surface of BA-0 could be divided into two regions, as circled in rock-salt phase E1 and layered phase E2, whereas le that of BA-7 is keeping the same layered phase (as circled in F1 and F2) from surface to bulk. The disappearance of rock-salt phase, which is also known as NiO in Ni-rich materials, explains the reduction of Ni^2+^ in Figure S2B. However, the *I*_(003)_/*I*_(104)_ values in Table S1 also exhibit that some of the modified materials suffer more severe cation mixing than BA-0, conflicting with the conclusions in Figure S2. Therefore, we put forward a hypothesis that weak acid such as BA has uneven leaching effect on the Ni-rich materials based on the results mentioned previously, which will be explored in the future.

There are several literatures pointing out that the materials will be more sensitive to the air/water after water washing (Xiong et al., 2013; Junhyeok et al., 2018). Hence, we also checked the storage performances of all the washed samples here. All of them were stored in the air for 15 days, and the relative humidity curve during store stage is shown in Figure S3. We renamed the samples after storage as BA-1-S–BA-9-S to distinguish the samples before from after storage. The TEM measurement was carried out first. As displayed in Figures S4A,B, the amount of impurity phase on the BA-0-S is much higher than BA-0, indicating that the RLC would easily form on the particles surface during storage. The TEM image of BA-7-S, one of the stored washed materials, is also placed in Figure S4C. There is only a little of amorphous impurities (which was marked by red circle) on the particle surface, suggesting the sensitivity of the treated samples to humid air had not been improved.

**Figure 5:**
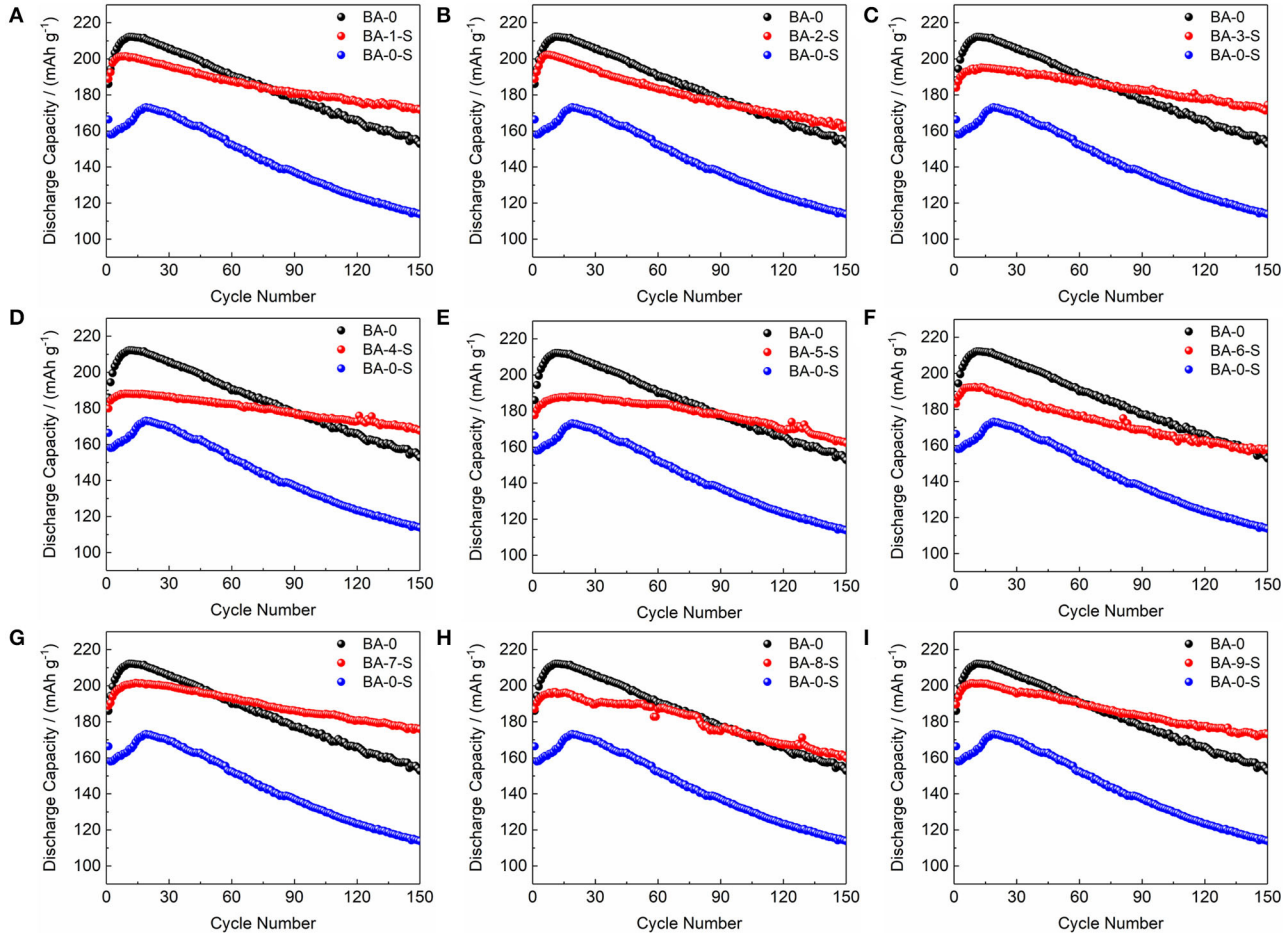
The comparisons of cycling performance of **(A–I)** BA-1-S to BA-9-S with BA-0 and BA-0-S at 0.2 C rate.

The electrochemical performances of stored materials, BA-1-S–BA-9-S, were tested as shown in Figure 5, Figure S5, and Table 2 (1 C = 200 mA/g). BA-1 and BA-0-S were also exhibited for comparisons. Figures S5A,C, Figure 5 point out that the BA-0 and BA-0-S have similar rapid drop tendency during cycling with a ~40 mAh/g difference, indicating an abrupt electrochemical performance degradation of Ni-rich material after storage. The first cycle voltage profiles of BA-0 and BA-0-S at 0.2 C rate are exhibited in Figure S5B, in which the BA-0-S owns a huge IR drop at discharge stage and a long constant voltage platform during charging. The huge IR drop comes from drastic parasitic reactions. Moreover, the charge capacity of all washed samples at constant voltage stage is shorter than BA-0-S as demonstrated on the bottom of Figure S5B, implying that the side reactions between RLC and electrolyte were suppressed after washing (Zhang et al., 2019). Figure 5 exhibits the detailed comparisons of stored treated materials with BA-0 and BA-S at 0.2 C cycling, which splits from Figure S5A. Even though the initial discharge capacities of all stored treated materials are lower than that of BA-0, their discharge capacity values are much larger than that of BA-0-S, indicating enhanced storage performances. The 150th capacity retentions after storing had increased from 68.39% of pristine material to 85.46–94.84% of the washed materials.

**Table 2:** Summary of the electrochemical performances of all the samples.

**Sample no**.	**Initial discharge capacity at 0.2 C**	**150th capacity retention at 0.2 C**	**Initial discharge capacity at 1 C**	**150th capacity retention at 1 C**
BA-0	190	80.42%	188.9	71.36%
BA-0-S	166.4	68.39%	167.3	59.83%
BA-1-S	188.9	90.95%	180.4	84.87%
BA-2-S	188.8	86.18%	183.1	83.67%
BA-3-S	184	94.84%	175.9	85.79%
BA-4-S	179.9	93.16%	184.3	86.00%
BA-5-S	177.7	91.33%	170.7	90.22%
BA-6-S	183.2	86.14%	183.3	79.71%
BA-7-S	188.8	93.22%	183.6	88.73%
BA-8-S	187.1	85.46%	177.6	85.25%
BA-9-S	189.6	91.24%	179.5	83.73%

**Figure 6:**
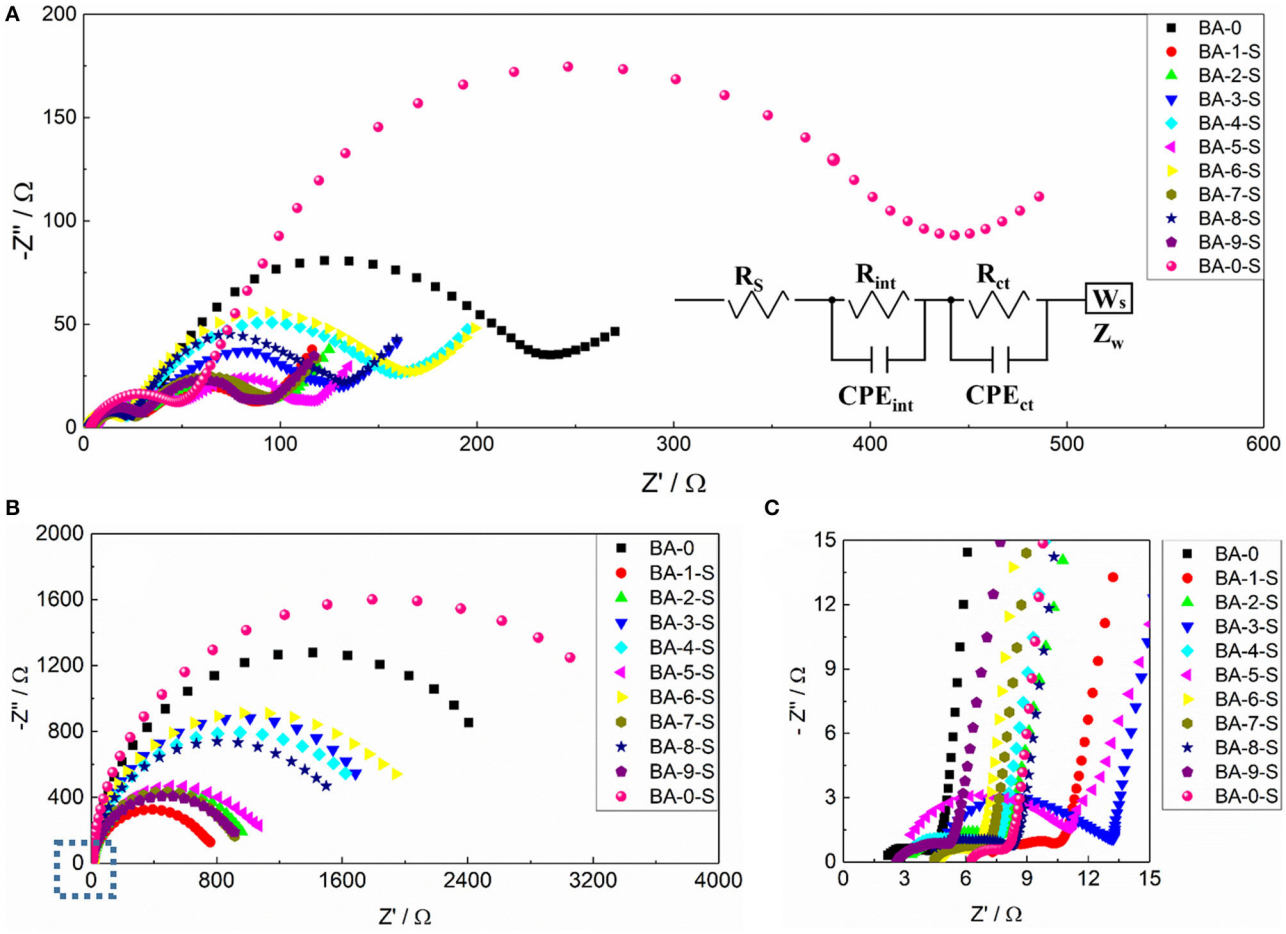
Nyquist plots of all samples after **(A)** 1st and **(B)** 100th cycles at 1 C/1 C rate and **(C)** the magnified area of marked area in **(B)**. The insert in **(A)** is the equivalent circuit model of all samples.

The side reactions between RLC and electrolyte will form a cathode–electrolyte interphase (CEI) film and influence the electrochemical kinetics (Grenier et al., 2017). The EIS analysis of all stored samples was applied after the 1st and 150th cycles to estimate the electrochemical kinetics changes, and the fresh BA-0 was also tested as a comparison. As the Nyquist plots shown in Figure 6, all curves consist of two semicircles and a line. The first semicircle at high frequency corresponds to the resistance of CEI film (*R*_int_), whereas the secondary one at intermediate frequency presents the charge transfer resistance (*R*_ct_) (Su et al., 2018a). All the samples exhibit different electrochemical kinetics even only after the first cycle (Figure 6A). BA-0-S owns the highest *R*_int_ and *R*_ct_ values among all samples, whereas the BA-0 owns the small *R*_int_ value but the second largest *R*_ct_ value. Because BA-0-S inherited from BA-0, it is easy to speculate that the large *R*_int_ values resulted from the RLC on the surface, which is formed during the storage. Benefiting from the reduced RLC amount, the washed samples exhibit better surface charge transfer, according to their smaller *R*_ct_ values than those of BA-0 and BA-0-S, no matter in 1st or 150th cycle. The low *R*_ct_ value of cathode material is in favor of its electrochemical performances (Su et al., 2018b), which is in accord with the better electrochemical performances of washed samples shown in Figure 5 and Table 2.

**Figure 7:**
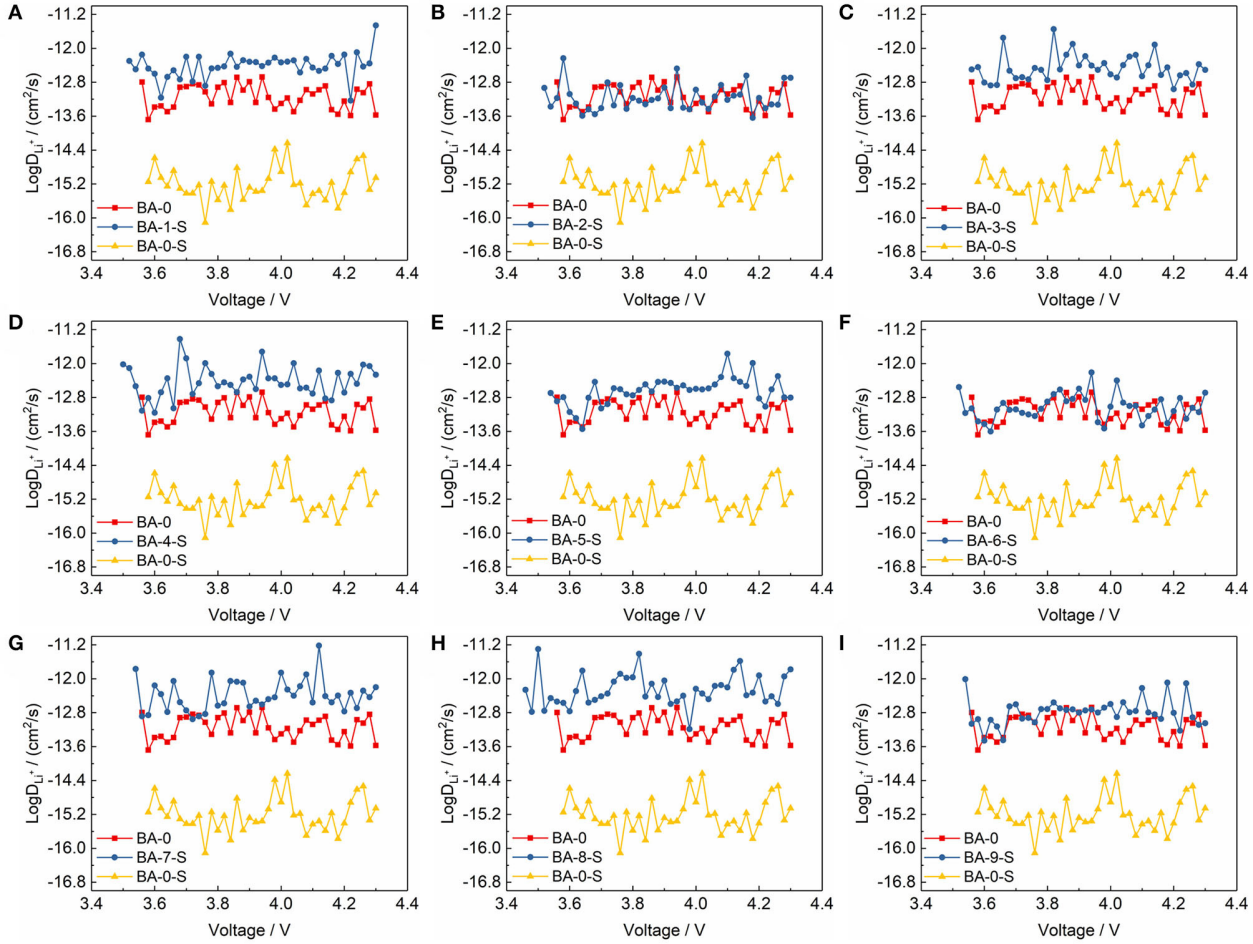
The comparisons of Li^+^ diffusion coefficients of **(A–I)** BA-1-S to BA-9-S with BA-0 and BA-0-S.

Not only the EIS, but also the PITT test, was performed to calculate the Li^+^ diffusion coefficient for analyzing the electrochemical kinetics of electrodes. The details of PITT test curves are shown in Figure S6, and the calculated Li^+^ diffusion coefficients are shown in Figure 7. We present three Li^+^ diffusion coefficient curves in Figures 7A–I, including one of stored washed materials, BA-0 and BA-0-S. The Li^+^ diffusion coefficient of BA-0-S is much smaller than BA-0 and all washed materials, which may ascribe to the block effect of RLC on the material surface. Meanwhile, the Li^+^ diffusion coefficient curves of BA-0 and all stored treated materials are similar, illustrating that the washing process will enhance the resistance of Ni-rich material to the air, suppressing the formation of RLC (Su et al., 2018a).

In conclusion, the positive effects of surface washing method *via* ethanol solvent with a little BA for Ni-rich LiNi_0.90_Co_0.06_Mn_0.04_O_2_ material have been proved, whereas the influences of washing parameters on the storage materials should also be evaluated. The analysis results of orthogonal factor level table, which were based on the capacity retention of stored materials at 0.2 C rate, are listed in Table 3. The ranking numbers of electrochemical result of all samples were defined from 1 to 9, and the material with higher capacity retention owns a lower number value. The I_L_,II_L_, and III_L_ values could be used to estimate the effects of levels A, B, and C on the corresponding factors. The *K*_1_, *K*_2_, and *K*_3_ values reflect the average rate of change, which equal to the values of I_L_/3, II_L_/3, and III_L_/3, respectively, in this work. *R* is the max–min values of corresponding K columns, reflecting the extent of effect of factors. According to the calculated estimates of the horizontal effects, we can figure out that the smaller solid–liquid ratio, shorter washing time, and higher concentration of BA would be better for the washing process. The optimal parameters are as follows: the solid–liquid ratio is 1 g/mL, the concentration of BA is 1 g/L, and the washing time is 5 min. Because the final pH values of all washed samples are still higher than 11.5, we can easily draw a conclusion that there are still lots of RLC on the surface of treated samples. The superfluous RLC means the whole washing process is incomplete, reflecting that more BA is needed in this treatment. With lower solid–liquid ratio and higher concentration of BA, the removal of RLC and undesired NiO phase would be more complete, which is a benefit for the electrochemical performances of Ni-rich materials. It is also worth noting that when prolonging the washing time, the positive effect was also achieved. With the largest *R* value in washing time column, it could be explained by the following reasons. When the washing time is short, the reaction products between BA and RLC would adhere on the surface of Ni-rich materials and transform into a thin layer of LiBO_2_ after calcination (Hu et al., 2017). The fast ionic conductor LiBO_2_ could not only improve the migration of Li^+^, but also protect the cathode material from the electrolyte erosion. Meanwhile, when the washing time become longer, the washing process would be thorough. The RLC and the reactants between BA and RLC, even some nanoparticles (Xu S. et al., 2019), would fell off from the surface of the secondary particles. Without the side reactions between RLC and small primary particles with electrolyte, the cycling stability of materials should be enhanced.

**Table 3:** Orthogonal factor level table of washed samples based on the electrochemical performance of stored materials at 0.2 C rate.

**Sample no**.	**Factors**			
	**Solid–liquid ratio (g/mL)**	**Washing time (min)**	**Concentration of BA (g/L)**	**Electrochemical results**
	**Level**			
	**A**	**B**	**C**	**No**.
BA-1	1	5	0.25	6
BA-2	1	10	0.5	7
BA-3	1	20	1	1
BA-4	5	5	1	3
BA-5	5	10	0.5	4
BA-6	5	20	0.25	8
BA-7	10	5	0.25	2
BA-8	10	10	1	9
BA-9	10	20	0.5	5
I_L_	14	11	16	*T* = 45
II_L_	15	20	16	
III_L_	16	14	13	
K_1_	4.67	3.67	5.33	
K_2_	5	6.67	5.33	
K_3_	5.33	4.67	4.33	
R	0.66	3	1	
Better level	A_1_	B_1_	C_3_	

## Conclusion

We proposed a low-cost surface washing method *via* ethanol solvent that contains low concentration of BA for LiNi_0.90_Co_0.06_Mn_0.04_O_2_ material. It has been determined that this treatment has the positive effects on removing the RLCs from the surface of Ni-rich cathode material. The fluid washing process could not only remove the RLC effectively, but also suppress the formation of RLC during the storage, leading to the superior storage electrochemical performances. The 150th capacity retentions after 15-day storing had increased from 68.39% of pristine material to 85.46–94.84% of treated materials. Besides, the effects of washing parameters such as solid–liquid ratio, washing time, and the concentration of BA to materials were also systematically studied. According to the orthogonal analysis, the suitable washing process needs smaller solid–liquid ratio, shorter washing time, and higher concentration of BA. This work provides a promising surface washing strategy for Ni-rich materials, which shows a great prospect in the industrial application.

## Data Availability Statement

The original contributions presented in the study are included in the article/Supplementary Material, further inquiries can be directed to the corresponding author/s.

## Author Contributions

YS, GC, and LC designed the experiment, characterized the samples, and wrote the manuscript. LL, CL, RD, and JL helped to the material synthesis. ZL contributed to the TEM test and corresponding analysis. YL, LB, GT, SC, and FW contributed to the data analysis and manuscript revision. All authors reviewed and approved the final submitted version of manuscript.

## Conflict of Interest

The authors declare that the research was conducted in the absence of any commercial or financial relationships that could be construed as a potential conflict of interest.
