# Supplementary material for: Clean the Ni-Rich Cathode Material Surface With Boric Acid to Improve Its Storage Performance
Source: Front Chem. 2020 Jul 24;8:573. doi: 10.3389/fchem.2020.00573 (PMC7393980; doi:10.3389/fchem.2020.00573)
Supplement: Supplementary file 1 [file Table_1.DOCX]

Supplementary Material

# Synthesis of LiNi_0.90_Co_0.06_Mn_0.04_O_2_ cathode material

The spherical hydroxide precursors were prepared by a traditional co-precipitation method. Transition-metal sulfates with NiSO_4_/CoSO_4_/MnSO_4_ = 90: 6: 4 (in molar ratio) were dispersed into deionized water and kept under stirring until a 2.0 mol/L solution was formed. The sodium hydroxide and ammonia mixing aqueous solution with a total concentration of 2.0 mol/L was prepared in the same way. All liquids were pumped into a 1 L volume continuous stirred tank reactor, and keeping the argon gas flow constant. The pH value and the reaction temperature were kept at 11 and 55℃, respectively. After 24 h of reaction, we got the green compacted precursors. The obtained precursor particles were mix with LiOH (1:1.05 in mol %) thoroughly. The LiNi_0.90_Co_0.06_Mn_0.04_O_2_ cathode material was got after mixture was precalcined for 5 h at 550 °C followed by 15 h at 720 °C under oxygen atmosphere.

# Supplementary Figures and Tables

## Supplementary Figures


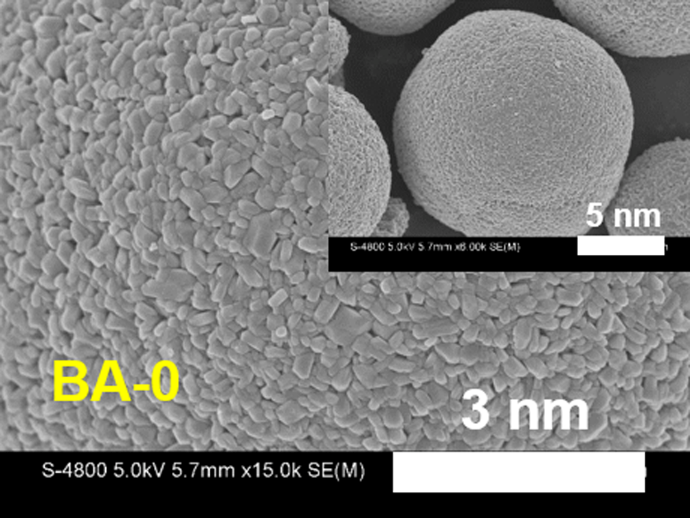


**Figure S1.** The SEM image of BA-0 sample.


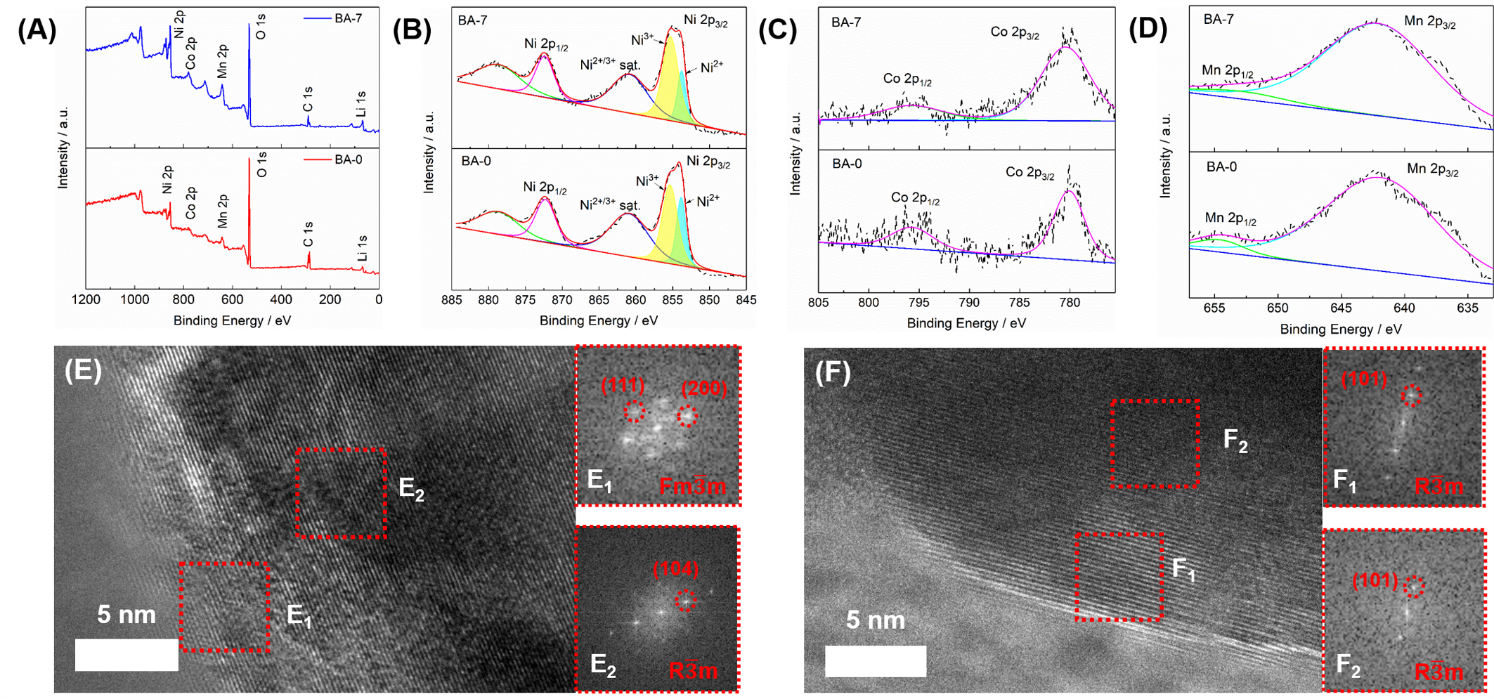


**Figure S2.** (A)-(D) The XPS patterns of BA-0 and BA-7. HR-TEM and FFT images of (D) BA-0 and (E) BA-7.


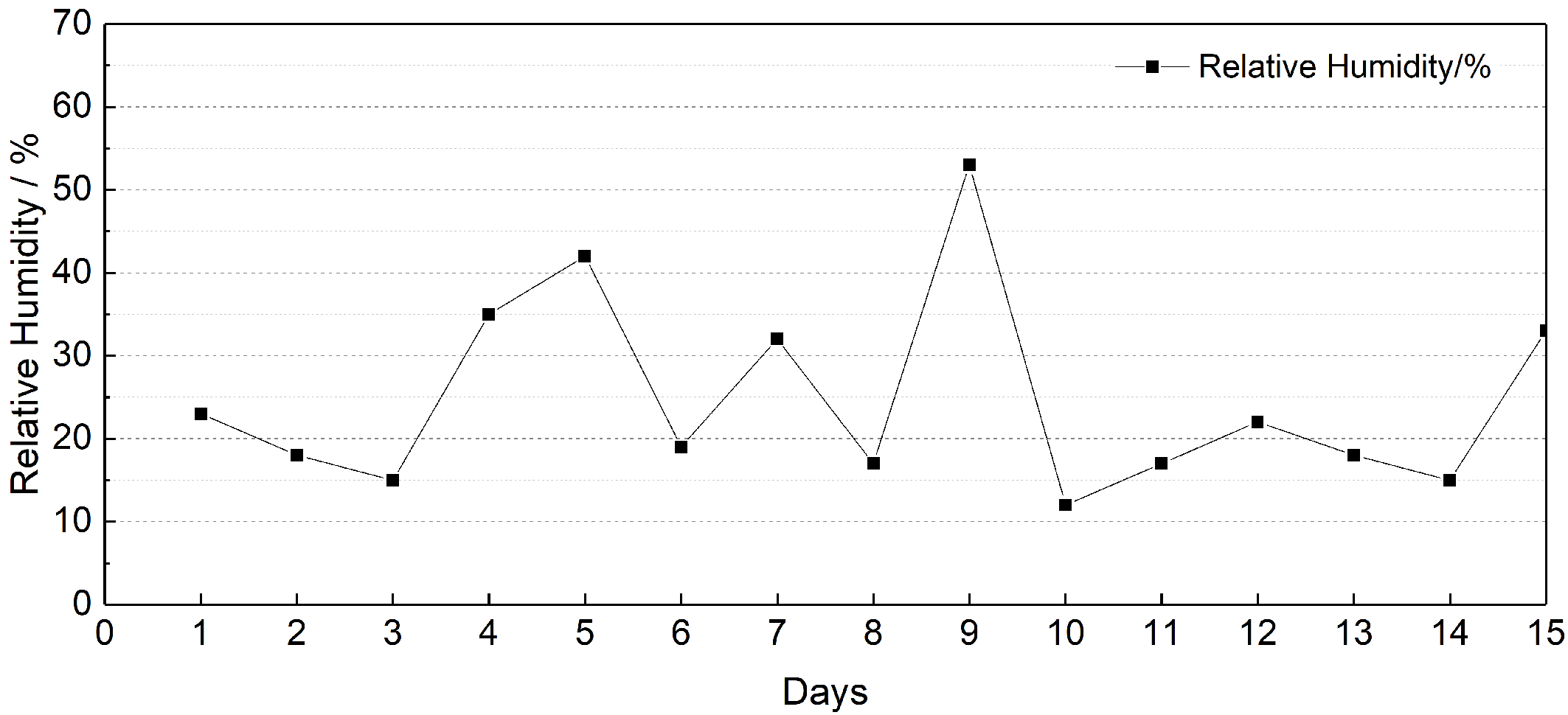


**Figure S3.** The relative humidity vs. storing time.


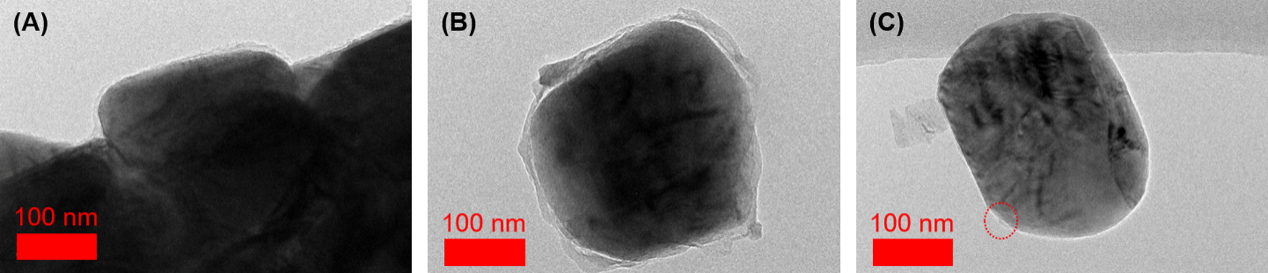


**Figure S4.** The TEM images of (A) BA-0, (B) BA-10 and (C) stored BA-7


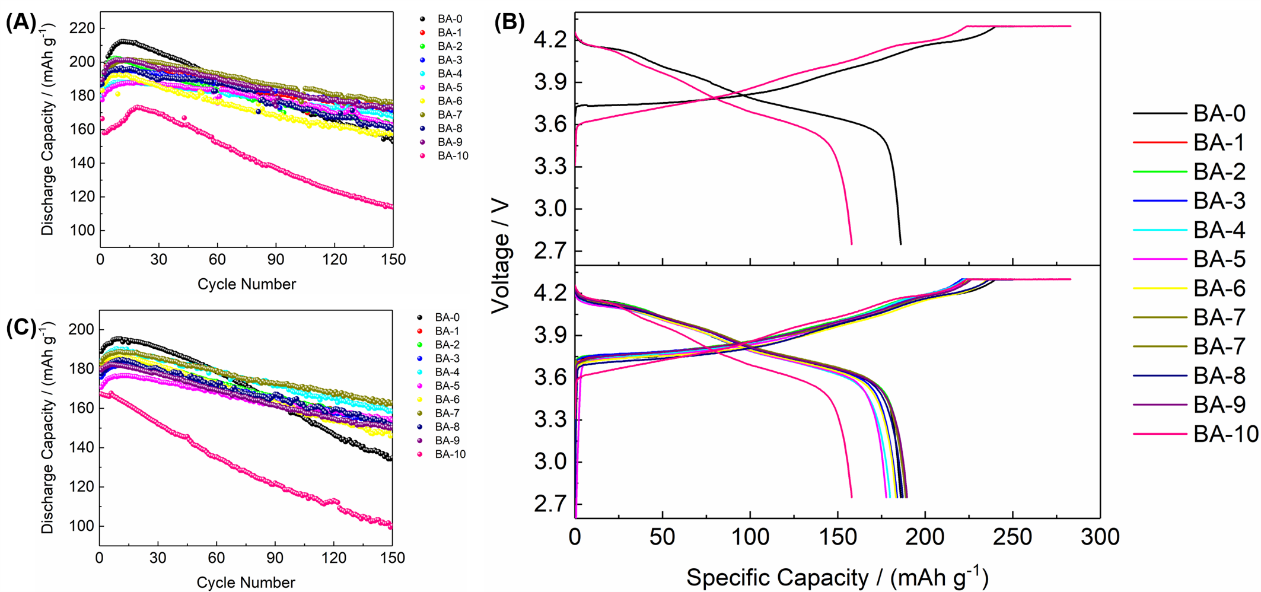


**Figure S5.** Electrochemical performances of all samples. (A) The cycling performance at 0.2C and (B) initial charge/discharge-voltage profiles at 0.2C; (C) cycling performance at 1C.


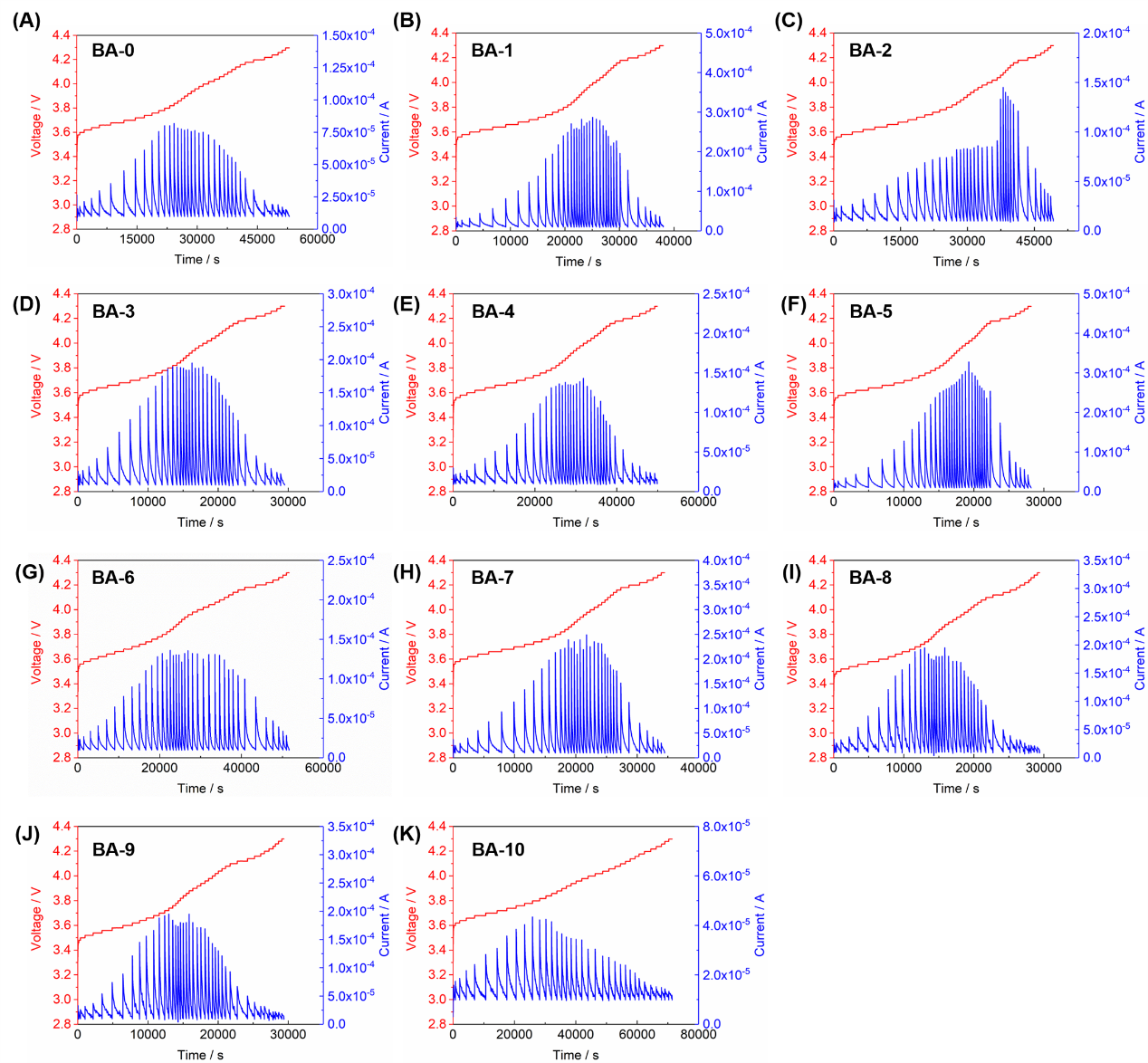


**Figure S6.** The details of PITT test for all samples.

## Supplementary Tables

Table S1. Calculated XRD results of all samples based on the patterns in Figure 2

| Samples | I_(003)_/I_(104)_ | Left-shift of I_(003)_ (2θ/ °) |
| --- | --- | --- |
| BA-0 | 1.42 | 0 |
| BA-1 | 1.47 | 0.16 |
| BA-2 | 1.43 | 0.16 |
| BA-3 | 1.41 | 0.18 |
| BA-4 | 1.39 | 0.12 |
| BA-5 | 1.40 | 0.16 |
| BA-6 | 1.50 | 0.14 |
| BA-7 | 1.48 | 0.14 |
| BA-8 | 1.36 | 0.08 |
| BA-9 | 1.47 | 0.14 |
